# Optimization of Islet Microencapsulation with Thin Polymer Membranes for Long-Term Stability

**DOI:** 10.3390/mi10110755

**Published:** 2019-11-06

**Authors:** Shota Toda, Artin Fattah, Kenta Asawa, Naoko Nakamura, Kristina N. Ekdahl, Bo Nilsson, Yuji Teramura

**Affiliations:** 1 Department of Bioscience and Engineering, College of Systems Engineering and Science, Shibaura Institute of Technology, Saitama 337-8570, Japan; bn15240@shibaura-it.ac.jp (S.T.); naoko@shibaura-it.ac.jp (N.N.); 2 Department of Immunology, Genetics and Pathology (IGP), Uppsala University, Dag Hammarskjölds väg 20, SE-751 85 Uppsala, Sweden; artinfattah@gmail.com (A.F.); kristina.nilsson_ekdahl@igp.uu.se (K.N.E.); bo.nilsson@igp.uu.se (B.N.); 3 Department of Bioengineering, School of Engineering, The University of Tokyo, 7-3-1 Hongo, Bunkyo-ku, Tokyo 113-8656, Japan; asawa@celly.t.u-tokyo.ac.jp; 4 Linnaeus Center of Biomaterials Chemistry, Linnaeus University, SE-391 82 Kalmar, Sweden

**Keywords:** microencapsulation, bioartificial pancreas, islet transplantation, polyethylene glycol-lipid (PEG-lipid), cell surface modification

## Abstract

Microencapsulation of islets can protect against immune reactions from the host immune system after transplantation. However, sufficient numbers of islets cannot be transplanted due to the increase of the size and total volume. Therefore, thin and stable polymer membranes are required for the microencapsulation. Here, we undertook the cell microencapsulation using poly(ethylene glycol)-conjugated phospholipid (PEG-lipid) and layer-by-layer membrane of multiple-arm PEG. In order to examine the membrane stability, we used different molecular weights of 4-arm PEG (10k, 20k and 40k)-Mal to examine the influence on the polymer membrane stability. We found that the polymer membrane made of 4-arm PEG(40k)-Mal showed the highest stability on the cell surface. Also, the polymer membrane did not disturb the insulin secretion from beta cells.

## 1. Introduction

Pancreatic islet transplantation has been examined for treating insulin-dependent diabetes mellitus (type I diabetes) [1]. Transplanted islets regulate the amount of released insulin in response to blood glucose concentration changes, thus providing nearly ideal control of glucose metabolism. Although the efficacy of islet transplantation has been proved in the treatment of diabetes patients, various issues including the side effects of immunosuppressive drugs and the donor shortage, still require resolution before the technique becomes a major treatment method [2,3,4]. The strong advantage is that islet transplantation is less invasive for patients since islet suspension is infused into the portal vein of patient’s liver which is a minor surgery in comparison to whole pancreas transplantation [5,6,7]. However, several donors are required to transplant sufficient numbers of islets, so that the patients can be free from insulin injections. Since immune reactions occur once the patients receive islets [8], those islets are gradually destroyed although immune-suppressive drugs are given to patients [2]. Therefore, a number of islets are transplanted to patients from several donors, which is a disadvantage. In addition, since donor shortage has been a serious problem in organ and cell transplantation [9], it would be valuable to solve this issue. Microencapsulated islets have been proposed as a bioartificial pancreas [10,11,12], and are expected to address the problem of immune rejection reactions. The bioartificial pancreas model involves the isolation of the islets from the host immune systems with polymer membranes such as a hydrogel. Low-molecular weight molecules such as insulin and nutrients can permeate through the polymer membranes while the attack from immunoglobulins and immune competent cells can be inhibited. In recent studies, alginate [13], a hybrid of poly ethylene glycol (PEG) and alginate [14], layer by layer of a poly ion complex or PEG [15,16,17,18] have been the materials mainly used for microcapsules. Particularly, alginate and agarose [19] hydrogels encapsulating islets have been successfully used to maintain normoglycemia in type 1 diabetes model animals. However, although the microencapsulated islets have been examined in clinical trials, the clinical outcomes are not the same as the results obtained with rodents; recipients continue to need exogenous insulin [20]. This result indicated that the number of transplanted microencapsulated islets was insufficient where there is no available transplantation site in the human body due to the total volume and size increase. Therefore, the size of microcapsules should be smaller; a thin polymer membrane is necessary to meet the clinical demand. Microencapsulation of islets using layer by layer (LBL) polymer membranes has been intensively studied [15,16,17]. Also, our group have studied islets microencapsulation with thin polymer membranes using polyethylene glycol conjugated phospholipid (PEG-lipid) and branched polymers [21]. The advantage of microencapsulation with thin polymer membrane is that it is possible to transplant those islets into portal vein of recipients, which is currently used as a transplantation site in clinical setting. Also, since there is no practical volume increase, we can transplant the sufficient number of islets whereas we cannot transplant enough number using hydrogels encapsulating islets. On the other hand, the stability of the thin polymer membrane has been unsolved issue. We also succeeded in the microencapsulation with thin polymer membrane. However, the stability of the polymer membrane remains a problem due to the cracking of the polymer membrane, which limits the use in clinical applications.

In this study, in an effort to enhance the membrane stability, we optimized the multiple-arm PEG polymer with different molecular weights for the formation of thin polymer membranes. Herein, we evaluated the long-term stability of the polymer membrane on the surface of erythrocytes. Also, a functional assay of the beta cells was performed after microencapsulation. 

## 2. Materials & Methods

### 2.1. Materials

*α*-*N*-Hydroxysuccinimidyl-*ω*-maleimidyl poly(ethylene glycol (NHS-PEG-Mal Mw: 5kDa), 1,2-dipalmitoyl-*sn*-glycerol-3-phosphatidylethanolamine (DPPE), hexaglycerol octa(mercaptoethyl) polyoxyethylene, (8-arm PEG-SH, PEG: MW of each arm: 20 kDa,) and pentaerythritol tetra((3-(3-maleimido-1-oxopropyl)amino)propyl)-polyoxyethylene, (4-arm PEG(40k)-Mal, 4-arm PEG(20k)-Mal, 4-arm PEG(10k)-Mal, PEG: MW of each arm: 40 kDa, 20kDa, 10kDa, respectively) were purchased from NOF Corporation (Tokyo, Japan). NaCl, MgCl_2_·6H_2_O, NaHCO_3_, Glucose, KCl, CaCl, D(+)-Glucose, and enzyme-linked immunosorbent assay (ELISA) kit for mouse insulin were purchased from FUJIFILM Wako Pure Chemical Corporation (Osaka, Japan). FITC-Conjugated bovine serum albumin (FITC-BSA), BSA, triethylamine, dichloromethane, and diethyl ether were purchased from Sigma-Aldrich Chemical Co. (St. Louis, MO, USA). Traut’s reagent, polyacrylamide spin desalting columns (spin column, 7K MWCO), fetal bovine serum (FBS) and penicillin-streptomycin, Liquid (P/S penicillin: 5000 IU/mL, streptomycin: 5000 µg/mL in 100 mL of 0.85% NaCl aqueous solution) were purchased from Thermo Fisher Scientific (Waltham, MA, USA). Anticoagulant Citrate Phosphate Dextrose Adenine Solution, (CPDA solution) and vacuum blood collection were purchased from TERUMO Corporation (Tokyo, Japan). Ethylenediaminetetraacetic acid solution, (EDTA, 0.5 M, pH 8.0,) and DMEM were purchased from Invitrogen (Carlsbad, CA, USA). Beta-TC-6 cells and CCRF-CEM (acute lymphocytic leukemia; human, ATCC CRL-8436) was purchased from American Type Culture Collection (ATCC, Manassas, VA, USA). 

### 2.2. Synthesize of Mal-PEG-Lipid

Maleimidyl poly(ethylene glycol)-conjugated phospholipid (Mal-PEG-lipid) was synthesized as previously reported [21,22]. Briefly, NHS-PEG-Mal (200 mg), triethylamine (50 mL), and DPPE (20 mg) were mixed in dichloromethane and stirred for 48 h at room temperature (RT) followed by the precipitation with diethyl ether. The precipitation was collected and dried, then Mal-PEG-lipid was obtained (190 mg, yield 80%).

### 2.3. Preparation of Human Erythrocytes from Human Whole Blood

Human whole blood was drawn from healthy volunteers. The whole blood was collected into 5 mL EDTA vacuum blood collection tube. After plasma and buffy coat were removed by centrifugation (15 min, 2500× *g*), the cells were resuspended in cold PBS containing 10 mM EDTA (EDTA/PBS). Then, the blood was centrifuged for 20 min at 200× *g* to remove platelets in the supernatant. This procedure was repeated six times. Finally, the number of erythrocytes was calculated by a cell counter (pocH-80i, SYSMEX, Kobe, Japan). When the platelet count is higher than 1.0 × 10^4^ cells/mL, the erythrocyte suspension was washed with EDTA/PBS to remove platelets completely. The experiments were approved by ethical committee of The University of Tokyo. 

### 2.4. Encapsulation of Human Erythrocytes with A Polymer Membrane

Human erythrocytes (200 µL, 1.6 × 10^9^ cells in 10 mM EDTA/PBS) were mixed with Mal-PEG-lipid (100 µL, 50 mg/mL in PBS) and incubated for 30 min at RT with gentle mixing. The erythrocyte suspension was placed on ice for 10 min and the suspension was added into 8-arm PEG-SH solution (100 µL, 5 mg/mL, in PBS, pH 7.4) with vigorous mixing. After the mixture was incubated for 10 min on ice, it was washed twice with PBS containing bovine serum albumin (BSA; 10 mg/mL) (BSA/PBS) by centrifugation. Then, a solution of 4-arm PEG-Mal (100 µL, 50 mg/mL, in PBS, pH 7.4) was mixed for 10 min on ice with gentle mixing, followed by washing with BSA/PBS twice. In order to increase the thickness of the polymer membrane, the above-mentioned procedure was repeated twice. The treated erythrocytes were reacted with Mal-PEG-lipid, 8-arm PEG-SH and then 4-arm PEG-Mal with the same conditions. Finally, the polymer membrane encapsulated erythrocytes were obtained.

For visualization of the polymer membrane on the surface of erythrocyte, FITC-BSA was used. After FITC-BSA (250 µL, 10 mg/mL in PBS) and Traut’s reagent (5.2 µL, 10 mg/mL in PBS) were mixed for 1hr at RT, FITC-BSA-SH was purified by spin column and diluted into 4 mg/mL with PBS. The FITC-BSA-SH (50 µL, 4 mg/mL in PBS) was added to the polymer membrane-coated erythrocytes and incubated for 5 min on ice. After washing with BSA/PBS twice, the treated erythrocytes were observed by confocal laser scanning microscopy (LSM510, Carl Zeiss, Jena, Germany) and analyzed by flow cytometry (BD LSR II, BD Biosciences, San Jose, CA, USA).

### 2.5. Long-Term Stability of Polymer Membrane on Erythrocytes

Three different PEG lengths of 4-arm PEG (10 kDa, 20 kDa, 40 kDa)-Mal were used for the microencapsulation of erythrocytes to examine the influence on the stability. Each erythrocyte was finally reacted with FITC-BSA-SH for the analysis by confocal laser scanning microscopy and flow cytometry. Encapsulated erythrocytes were incubated in a CPDA solution at 37 °C for 44 days at 5% CO_2_. 

### 2.6. Microencapsulation of Mouse Beta Cells with a Polymer Membrane

Beta-TC-6 cells (Cell line of beta cells) were cultured in 10 mL DMEM medium supplemented with 15% fetal bovine serum, 50 U/mL penicillin, and 50 µg/mL streptomycin. For the microencapsulation of beta cells, they were treated by the same procedure as above described for erythrocyte. The used cell number was 4.0 × 10^6^ cells. For the visualization, polymer membrane-encapsulated beta cells were treated with FITC-BSA-SH (30 µL, 4 mg/mL in PBS). The fluorescence images of all samples were observed using confocal laser scanning microscopy and analyzed by flow cytometry.

### 2.7. Glucose-Responsive Insulin Secretion of Membrane-Encapsulated Beta Cells

Insulin secreting ability of membrane-encapsulated beta-TC-6 cells was evaluated using a static system. Beta-TC-6 cells without any treatment were used as a control. Beta cells were incubated with Krebs-Ringer buffer (KRB) supplemented with 33 mM glucose for 1 h at 37 °C. KRB was prepared as follows: NaCl (702 mg, Wako), MgCl_2_·6H_2_O (22 mg), NaHCO_3_ (210 mg), Glucose (300 mg), KCl (37.2 mg), CaCl (27.8 mg) and BSA (100 mg) were added to pure water (100 mL). After pH of the solution was adjusted to pH 7.4, the solution was sterilized using a membrane filter. The concentration of mouse insulin was measured using an ELISA kit.

### 2.8. Statistical Analysis

Results are presented as mean ± SD. Data plotting and statistical analysis were performed using GraphPad Prism Version 6.0 (GraphPad Software, San Diego, CA, USA) for Macintosh software. 

## 3. Results

### 3.1. Encapsulation of Erythrocytes with Polymer Membrane

**Figure 1:**
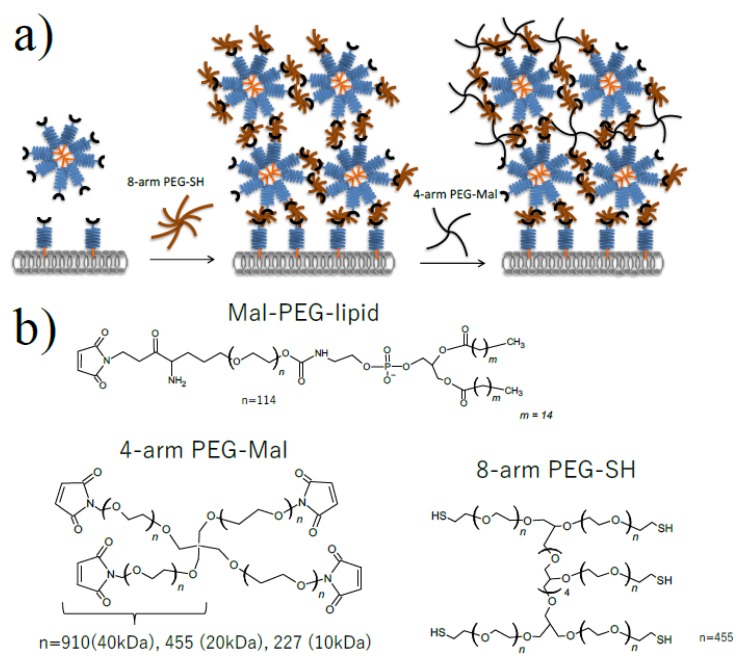
Microencapsulation of cells using Mal-PEG-lipid, 4-arm PEG-Mal and 8-arm PEG-SH. (**a**) Schematic illustration of cell microencapsulation (**b**) Chemical structure of Mal-PEG-lipid, 4-arm PEG(10k, 20k, 40k)-Mal and 8-arm PEG(20 kDa)-SH.

**Figure 2:**
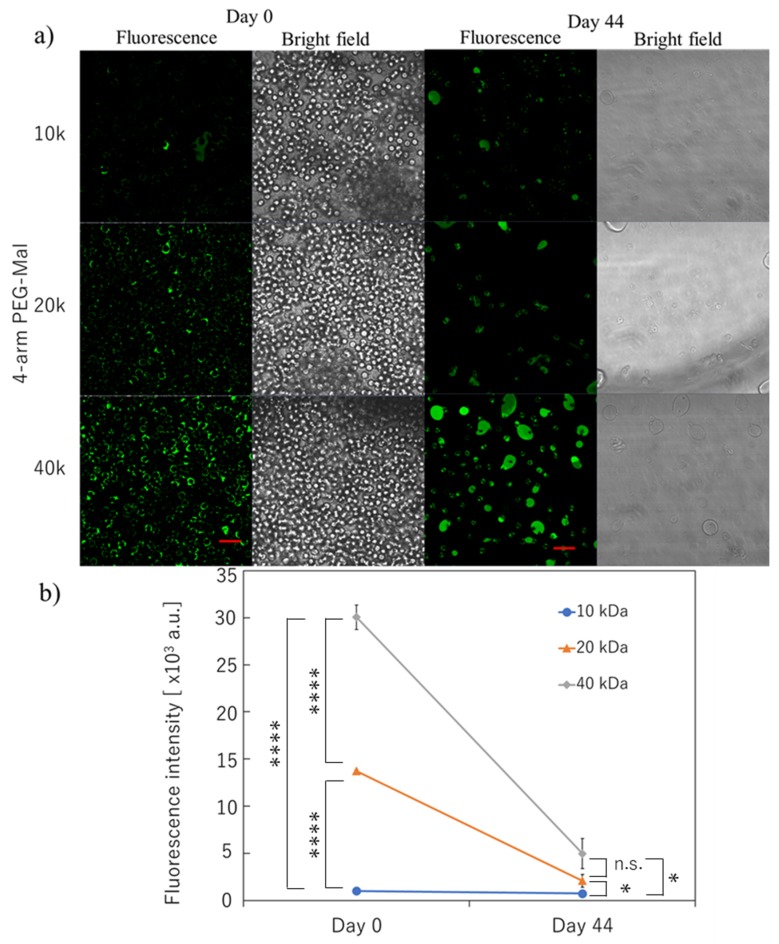
Microencapsulation of human erythrocytes with different 4-arm PEG-Mal. (**a**) Confocal microscopic images of microencapsulation of human erythrocytes with 4-arm PEG(10k, 20k, and 40k)-Mal. FITC-BSA-SH was used for the visualization of the polymer membrane. Scale bar: 20 µm. (**b**) Quantitative analysis of polymer membrane on human erythrocytes using flow cytometry at 0 and 44 days. Error bars indicate standard deviation; n = 3. * = *p* < 0.05; **** = *p* < 0.0001; n.s. = not significant.

By combining PEG-lipid and multiple PEG chain-branched polymers (Figure 1), we tried to form a stable polymer membrane on the surface of erythrocytes. We decided to use erythrocytes for the microencapsulation since these cells do not proliferate, and it is appropriate to evaluate the membrane stability quantitatively. Also, since the erythrocyte is very fragile, it can be easily damaged and destroyed if the cellular membrane is influenced by the surface modification. Therefore, it is a suitable model for cytotoxicity. The fluorescence signal was from FITC-BSA, which was covalently immobilized on the polymer membrane where FITC-BSA-SH was conjugated to the 4-arm PEG-Mal. From the confocal microscopic observation, a thick polymer membrane could be formed on the surface of erythrocytes (Figure 2a). The membrane thickness was thicker than a single polymer coating with PEG-lipid, and the thickness of the membrane was at the micro-meter level. Based on the analysis of flow cytometry, the fluorescence intensity of the polymer membrane depended on the molecular weight of PEG of 4-arm PEG-Mal; the fluorescence intensity increased with increasing molecular weight of PEG where the fluorescence intensity of 4-arm PEG(40k)-Mal membrane was 3.0 × 10^4^ whereas the values were 1.4 × 10^4^ and 1.0 × 10^3^ for 4-arm PEG(20k)-Mal and 4-arm PEG(10k)-Mal membranes, respectively. This result indicates that thicker polymer membranes could be formed on the erythrocyte surface by using the higher molecular weight variant of the 4-arm PEG-Mal. 

The long-term stability of the polymer membrane on the erythrocytes was also evaluated. As indicated in Figure 2a, the polymer membrane could be still detected on the surface of erythrocyte for the three conditions at 44 days. Approximately 50% of erythrocytes were lysed during incubation over a long time even though they were incubated in CPDA solution. However, there were still encapsulated erythrocytes left for the analyses, where the number of cells was comparable to that of non-modified erythrocytes. Some encapsulated erythrocytes aggregated to show large capsules surrounding erythrocytes during the incubation. We observed strong fluorescence from the polymer membrane using 4-arm PEG(40k)-Mal, indicating the stable polymer membrane was on the erythrocytes. The quantification of the fluorescence intensity from each cell was also demonstrated using flow cytometry; the fluorescence intensity of 4-arm PEG(40k)-Mal membrane was 5.0 × 10^3^ whereas the intensities were 2.1 × 10^3^ and 7.0 × 10^2^ for 4-arm PEG(20k)-Mal and 4-arm PEG(10k)-Mal membranes, respectively. The results indicated that the polymer membrane encapsulation using 40k PEG-Mal showed the highest stability for the long-term stability of membrane encapsulation.

### 3.2. Encapsulation of Mouse Beta-Cells

**Figure 3:**
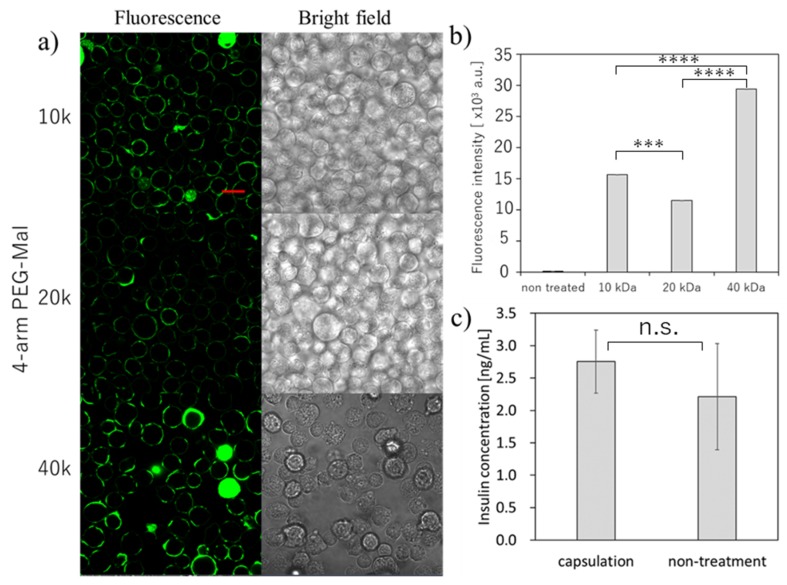
Microencapsulation of beta-TC-6 cells. with different 4-arm PEG-Mal. (**a**) Confocal microscopic images of microencapsulation of beta-TC-6 cells with 4-arm PEG (10k, 20k, and 40k)-Mal. FITC-BSA-SH was used for the visualization of the polymer membrane. Scale bar: 20 µm. (**b**) Quantitative analysis of polymer membranes on beta-TC-6 cells using flow cytometry. Error bars indicate standard deviation; n = 3. (**c**) Glucose-responsive insulin secretion from microencapsulated and non-treated beta-TC-6 cells measured by ELISA. Error bars indicate standard deviation; n = 3. *** = *p* < 0.001; **** = *p* < 0.0001; n.s. = not significant.

Then, we tried the microencapsulation of beta cells and examined the insulin secretion ability of the beta cells (Figure 3). Here, we also used the three different forms of 4-arm PEG(10k, 20k, 40k)-Mal for the optimization. As shown in Figure 3a, we could successfully perform the microencapsulation of beta cells using all three 4-arm PEG-Mal. The thicker polymer membrane was observed as seen in erythrocytes, and the fluorescence intensity from the polymer membrane using 4-arm PEG(40k)-Mal was the highest (Figure 3a,b). The tendency was similar as the results observed in erythrocytes. The secretion of insulin was evaluated for polymer membrane encapsulated beta cells (Figure 3c). The concentration of insulin from the encapsulated and non-treated cells was 2.75 ± 0.49 and 2.21 ± 0.82 ng/mL, respectively, values which showed no significant difference (*p* = 0.47). The beta cells encapsulated within polymer membrane responded to the change of glucose level and the insulin secretion was almost equal between the polymer membrane encapsulated beta cells and non-encapsulated beta cells, indicating that the polymer membranes have semi-permeability and beta cell function was not affected by the polymer membrane encapsulation.

## 4. Discussion

Microencapsulation of islets offers several advantages. Patients can be free from immune-suppressive drugs because of its immune-isolation property. In addition, not only human islets, but also porcine islets can be transplanted into human patients [23,24]. Since the donor shortage is a serious problem in islet transplantation, it would be beneficial for patients to use porcine islets as a resource. However, more immune rejection reactions take place in xenogeneic transplantation compared to allogeneic transplantation [25]. Therefore, the recipients would still need more immune suppressive therapy, which would be generally difficult for them. One approach to avoid this issue is microencapsulation of porcine islets. In fact, some clinical trials have been conducted using porcine islets which were encapsulated by alginate hydrogel [24,26]. The transplantation site is the intraperitoneal cavity. However, the clinical outcomes were not satisfactory because patients could not be free of insulin injection even after transplantation with microcapsule of porcine islets. This result suggested that total number of transplanted microcapsules of porcine islets were not sufficient to control patients’ glucose metabolism. Low oxygen supply [27,28] and fibrosis [29] can also affect the islet survival. Another important point is that patients cannot receive an adequate number of microcapsules due to the volume increase and large size of the capsules [18]. There is not enough physical space for the transplantation in human body. Therefore, polymer membrane-based microencapsulation will be a promising approach to solve these issues. However, the low membrane stability, where the polymer membrane detaches from the cell surface, is a challenge that remains to be solved. 

In this study, we tried to form thin and stable polymer membranes on the cell surface by a method slightly modified from our previous report [21]. Our approach is based on the layer-by-layer polymer deposition and involving Mal-PEG-lipid micelles where thick PEG membranes can be formed by combining with the micelles in single step. Also, the cross linking by 4-arm PEG-Mal makes it possible to form covalent bonding in the membrane, which can be a more stable linkage. By using 4-arm PEG-Mal, it was possible to form several LBL membranes by covalent bonding after repeating the same procedure, resulting in the increase of the membrane thickness and uniform coating. However, we did not optimize spacer length of 4-arm PEG-Mal for the stable polymer membrane. Here, we focused on 4 arm PEG-Mal and examined the different lengths of spacer from 10k to 40k PEG to increase the stability and the uniformity of the polymer membrane on the cellular surface. Also, since we did not try the single cell microencapsulation of insulin secreting cells so far. The long-term stability of the membrane on erythrocytes was evaluated because there is no cell division in these model erythrocytes, and the insulin secretion was studied using beta-TC-6 cells. In our method, thin polymer membranes could be formed only on the cell surface and also the membrane is covalently cross-linked, which makes it more stable. We found that the formed polymer membrane could be maintained for 44 days, which is more stable than a single polymer coating. And we also revealed that longer 4-arm PEG-Mal (40k) contributed to improvement of the stability and thickness of the membrane where the longer PEG spacer effectively formed cross links among SH groups in the membrane. In fact, the polymer membrane using 4-arm PEG(40k)-Mal is more stable than the polymers using 4-arm PEG(10k and 20k)-Mal. In addition, microencapsulated beta-TC-6 cells using 4-arm PEG-Mal (40k) showed insulin released, which indicates that the cells could respond and release insulin through the polymer membranes.

## 5. Conclusions

Thin polymer membranes can be formed on the cell surface using Mal-PEG-lipid and branched PEG without influence on the cellular membrane structure where Mal-PEG-lipid is used as an anchor and the micelle is also used a cross-linker for the polymer membranes. Therefore, it was possible to fabricate substantially thick membrane on each cell, which was different from layer-by-layer polymer deposition approach. The membrane thickness can be controlled by the repeating procedure, and the membrane stability can be improved by combination with a longer spacer of the branched polymers as well. The membrane stability is lower than hydrogel-based capsules because the membrane uniformity is still poor and the membrane is broken from the lack. However, we will continue to work on the improvement of the stability. In future, we will try our technique to transplant not only islets but also mesenchymal stem cells (MSCs) for model animals. Since our approach is available for single cell coating, we can inject encapsulated MSCs intravenously for the therapeutic purpose.

## References

[1] James Shapiro A.M., Lakey J.R.T., Ryan E.A., Korbutt G.S., Toth E., Warnock G.L., Kneteman N.M., Rajotte R.V. (2000). Islet transplantation in seven patients with type 1 diabetes mellitus using a glucocorticoid-free immunosuppressive regimen. N. Engl. J. Med..

[2] Ryan E.A., Paty B.W., Senior P.A., Bigam D., Alfadhli E., Kneteman N.M., Lakey J.R.T., James Shapiro A.M. (2005). Five-Year Follow-Up After Clinical Islet Transplantation. Diabetes.

[3] Calafiore R., Basta G., Luca G., Lemmi A., Montanucci M.P., Calabrese G. (2006). Microencapsulated Pancreatic Islet Allografts into Nonimmunosuppressed Patients with Type 1 Diabetes. Diabetes Care.

[4] James Shapiro A.M., Pokrywczynska M., Ricordi C. (2017). Clinical pancreatic islet transplantation. Nat. Rev. Endocrinol..

[5] Moassesfar S., Masharani U., Frassetto L.A., Szot G.L., Tavakol M., Stock P.G., Posselt A.M. (2016). A Comparative Analysis of the Safety, Efficacy, and Cost of Islet Versus Pancreas Transplantation in Nonuremic Patients with Type 1 Diabetes. Am. J. Transplant..

[6] Frank A., Deng S., Huang X., Velidedeoglu E., Bae Y.-S., Liu C., Abt P., Stephenson R., Mohiuddin M., Thambipillai T. (2004). Transplantation for Type I Diabetes: Comparison of Vascularized Whole-Organ Pancreas with Isolated Pancreatic Islets. Ann. Surg..

[7] Maffi P., Scavini M., Socci C., Piemonti L., Caldara R., Gremizzi C., Melzi R., Nano R., Orsenigo E., Venturini M. (2011). Risks and Benefits of Transplantation in the Cure of Type 1 Diabetes: Whole Pancreas Versus Islet Transplantation. A Single Center Study. Rev. Diabet. Stud..

[8] Evgenov N.V., Medarova Z., Pratt J., Pantazopoulos P., Leyting S., Bonner-Weir S., Moore A. (2006). In Vivo Imaging of Immune Rejection in Transplanted Pancreatic Islets. Diabetes.

[9] Girlanda R. (2016). Deceased organ donation for transplantation: Challenges and opportunities. World J. Transplant..

[10] Iacovacci V., Ricotti L., Menciassi A., Dario P. (2016). The bioartificial pancreas (BAP): Biological, chemical and engineering challenges. Biochem. Pharmacol..

[11] Desai T., Shea L.D. (2017). Advances in islet encapsulation technologies. Nat. Rev. Drug Discov..

[12] Sakata N., Sumi S., Yoshimatsu G., Goto M., Egawa S., Unno M. (2012). Encapsulated islets transplantation: Past, present and future. World J. Gastrointest. Pathophysiol..

[13] Dufrane D., Rose-Marie G., Alain S., Yves G., Pierre G. (2006). Six-Month Survival of Microencapsulated Pig Islets and Alginate Biocompatibility in Primates: Proof of Concept. Transplantation.

[14] Villa C., Manzoli V., Abreu M.M., Verheyen C.A., Seskin M., Najjar M., Molano R.D., Torrente Y., Ricordi C., Tomei A.A. (2017). Effects of Composition of Alginate-Polyethylene Glycol Microcapsules and Transplant Site on Encapsulated Islet Graft Outcomes in Mice. Transplantation.

[15] Syed F., Bugliani M., Novelli M., Olimpico F., Suleiman M., Marselli L., Boggi U., Filipponi F., Raffa V., Krol S. (2018). Conformal coating by multilayer nano-encapsulation for the protection of human pancreatic islets: In-vitro and in-vivo studies. Nanomed. Nanotechnol. Biol. Med..

[16] Pathak S., Pham T.T., Jeong J.-H., Byun Y. (2019). Immunoisolation of pancreatic islets via thin-layer surface modification. J. Control. Release.

[17] Haque M.R., Kim J., Park H., Lee H.S., Lee K.W., Al-Hilal T.A., Jeong J.-H., Ahn C.-H., Lee D.S., Kim S.J. (2017). Xenotransplantation of layer-by-layer encapsulated non-human primate islets with a specified immunosuppressive drug protocol. J. Control. Release.

[18] Miura S., Teramura Y., Iwata H. (2006). Encapsulation of islets with ultra-thin polyion complex membrane through poly(ethylene glycol)-phospholipids anchored to cell membrane. Biomaterials.

[19] Iwata H., Takagi T., Amemiya H., Shimizu H., Yamashita K., Kobayashi K., Akutsu T. (1992). Agarose for a bioartificial pancreas. J. Biomed. Mater. Res..

[20] Tuch B.E., Keogh G.W., Williams L.J., Wu W., Foster J.L., Vaithilingam V., Philips R. (2009). Safety and Viability of Microencapsulated Human Islets Transplanted into Diabetic Humans. Diabetes Care.

[21] Teramura Y., Oommen O.P., Olerud J., Hilborn J., Nilsson B. (2013). Microencapsulation of cells, including islets, within stable ultra-thin membranes of maleimide-conjugated PEG-lipid with multifunctional crosslinkers. Biomaterials.

[22] Teramura Y., Kaneda Y., Iwata H. (2007). Islet-encapsulation in ultra-thin layer-by-layer membranes of poly(vinyl alcohol) anchored to poly(ethylene glycol)-lipids in the cell membrane. Biomaterials.

[23] Gazda L.S., Collins J., Lovatt A., Holdcraft R.W., Morin M.J., Galbraith D., Graham M., Laramore M.A., Maclean C., Black J. (2016). A comprehensive microbiological safety approach for agarose encapsulated porcine islets intended for clinical trials. Xenotransplantation.

[24] Elliott R.B., Escobar L., Tan P.L.J., Muzina M., Zwain S., Buchanan C. (2007). Live encapsulated porcine islets from a type 1 diabetic patient 9.5 yr after xenotransplantation. Xenotransplantation.

[25] Elliott R.B. (2011). Towards xenotransplantation of pig islets in the clinic. Curr. Opin. Organ Transplant..

[26] Matsumoto S., Tan P., Baker J., Durbin K., Tomiya M., Azuma K., Doi M., Elliott R.B. (2014). Clinical Porcine Islet Xenotransplantation Under Comprehensive Regulation. Transplant. Proc..

[27] Barkai U., Weir G.C., Colton C.K., Ludwig B., Bornstein S.R., Brendel M.D., Neufeld T., Bremer C., Leon A., Evron Y. (2013). Enhanced Oxygen Supply Improves Islet Viability in a New Bioartificial Pancreas. Cell Transplant..

[28] Lehmann R., Zuellig R.A., Kugelmeier P., Baenninger P.B., Moritz W., Perren A., Clavien P.-A., Weber M., Spinas G.A. (2007). Superiority of Small Islets in Human Islet Transplantation. Diabetes.

[29] Strand B.L., Ryan L., In’t Veld P., Kulseng B., Rokstad A.M., Skjåk-Bræk G., Espevik T. (2001). Poly-L-Lysine Induces Fibrosis on Alginate Microcapsules via the Induction of Cytokines. Cell Transplant..

